# Clinical outcomes of upfront combination therapy for portopulmonary hypertension^☆^

**DOI:** 10.1016/j.ijcrp.2024.200294

**Published:** 2024-05-31

**Authors:** Takatoyo Kiko, Ryotaro Asano, Hiroyuki Endo, Naruhiro Nishi, Hiroya Hayashi, Jin Ueda, Tatsuo Aoki, Akihiro Tsuji, Takeshi Ogo

**Affiliations:** a Division of Pulmonary Circulation, Department of Cardiovascular Medicine, National Cerebral and Cardiovascular Center, Japan; b Department of Vascular Physiology, National Cerebral and Cardiovascular Center Research Institute, Japan

**Keywords:** Portopulmonary hypertension, Upfront combination therapy, Pulmonary hypertension

## Abstract

**Background:**

Limited data exists on upfront combination therapy for portopulmonary hypertension. We evaluated the clinical efficacy, long-term outcomes, and safety of upfront combination therapy in patients with portopulmonary hypertension.

**Methods:**

We performed a retrospective, single-center cohort study involving a final analysis of 33 consecutive patients diagnosed with portopulmonary hypertension who were taking pulmonary arterial hypertension-specific medication. We compared hemodynamic parameters, risk profiles, composite clinical worsening events, and safety between monotherapy (n = 23) and upfront combination therapy (n = 10).

**Results:**

Twenty-seven patients (82 %) were classified into the Child–Pugh A stage. The change ratios of pulmonary vascular resistance (−32 % vs. −57 %, P = 0.006) were significantly better with upfront combination therapy. Upfront combination therapy also showed significant improvement in risk profiles. Kaplan–Meier analysis showed that the composite event-free rate was significantly lower in patients who received upfront combination therapy than in those who received monotherapy (P = 0.016), although no statistical differences were observed in all-cause death. In the univariate Cox proportional hazards analysis, upfront combination therapy was a factor for decreasing composite clinical worsening outcomes (hazard ratio 0.190, 95 % confidence interval 0.042–0.854; P = 0.030). No significant hepatic impairments were observed over 2 years of follow-up in the upfront combination group.

**Conclusions:**

In patients with portopulmonary hypertension, upfront combination therapy significantly improved symptoms and short-term hemodynamics, and reduced long-term clinical worsening events without serious adverse effects. This study's findings suggest that patients with portopulmonary hypertension presenting with mild hepatic impairment benefit from upfront combination therapy.

## 1 Introduction

Portopulmonary hypertension (PoPH) is a rare form of pulmonary arterial hypertension (PAH) that is secondary to portal hypertension or portosystemic shunt, making up only 5–15 % of all cases of PAH [[1], [2], [3], [4]]. The underlying pathophysiology of PoPH shares similarities with idiopathic PAH, including smooth muscle cell proliferation, plexiform lesions, vasoconstriction, and *in situ* thrombosis. Given the relative rarity of PoPH, treatment strategies and long-term survival remain limited.

Recent studies have shown that PAH-specific treatment is potentially beneficial for the treatment of PoPH. In the largest group of patients with PoPH examined, monotherapy with endothelin receptor antagonists (ERA) improved hemodynamics [5,6]. Although the use of pulmonary vasodilators requires caution in patients with hepatic impairment [7], monotherapy seems to have acceptable safety. Patients with PoPH experience a delay in the initiation of treatment and a greater tendency towards monotherapy [8].

The benefits of upfront combination therapy for PoPH when compared to monotherapy are not well known. Upfront combination therapy targeting PAH may have improved therapeutic efficacy in patients with PAH, as evidenced by a decreased risk of worsening clinical symptoms and a probable improvement of exercise capacity and hemodynamics [9,10]. The efficacy and safety of PAH-specific treatment, particularly for combination PAH treatment in PoPH, remain unknown. This may be due to most clinical trials on the safety of PAH medication excluding patients with PoPH, differences in pathological condition [11], and a paucity of studies on combination therapy for PoPH [3,12]. The present study aimed to preliminarily elucidate the clinical and hemodynamic efficacy, long-term clinical outcomes, and safety of upfront combination therapy compared to monotherapy in patients with PoPH.

## 2 Methods

### 2.1 Patient selection

This single-center, retrospective study enrolled 51 consecutive patients with PoPH who were admitted to the National Cerebral and Cardiovascular Center between November 1997 and May 2023. The patient flowchart is shown in Supplemental Fig. 1. From the total, 18 patients were excluded because of a lack of data or absence of PAH-specific medication. The remaining 33 patients, who were specifically prescribed PAH-specific medication, were included in the analysis. The diagnosis of PoPH was based on the presence of otherwise unexplained pre-capillary pulmonary hypertension in patients with portal hypertension or a portosystemic shunt, diagnosed based on standard criteria [13]. PAH was confirmed by right heart catheterization (RHC) with a mean pulmonary artery pressure (PAP) ≥ 25 mm Hg and a pulmonary artery wedge pressure (PAWP) ≤ 15 mm Hg. Study inclusion required pulmonary vascular resistance (PVR) of 3 Wood units or more. Clinical assessments were performed simultaneously, and included clinical data, brain natriuretic peptide (BNP) levels, serum biomarkers, and 6-min walk distance (6 MWD). The cardiac index was determined using the indirect Fick method and corrected for the body surface area, and the PVR was calculated. Upfront combination therapy was defined as treatment with at least two PAH drugs (including ERA, phosphodiesterase type 5 inhibitors, or soluble guanylate cyclase stimulator) administered simultaneously. The study protocol was approved by the ethics committee of the National Cerebral and Cardiovascular Center (R20075).

### 2.2 Measurements

We evaluated the RHC and clinical data simultaneously before the initiation of upfront combination therapy after at least a three-month follow-up time. We also evaluated changes in risk using the simplified four-strata risk-assessment tool, based on refined cutoff levels for the World Health Organization (WHO) functional class, 6 MWD results, and BNP levels [13,14]. The low-risk criteria for the French invasive risk assessment approach were defined as 6 MWD >440 m, WHO functional class I or II, right atrial pressure <8 mm Hg, and cardiac index ≥2.5 L/min/m^2,^ [15,16]. REVEAL 2.0 Lite score was evaluated as previously described [17].

The endpoint in the time-to-event analysis was a composite of death and clinical events related to PAH from the time PAH-specific medicine was started, as in previous studies [10,18,19]. Clinical events related to PAH included disease progression or worsening that resulted in hospitalization, initiation of parenteral prostanoid therapy or long-term oxygen therapy, or the need for lung transplantation, as judged by a physician. Disease progression was defined as a decrease from the baseline of the 6-min walk distance of at least 15 % accompanied by a worsening of the WHO functional class or the need for additional PAH-specific medication due to worsening PH.

### 2.3 Statistical analysis

Continuous variables are presented as means ± standard deviation or median deviation (interquartile range). Differences in continuous variables between the two groups were compared using Student's t-test, the Wilcoxon rank-sum test, or the Mann–Whitney *U* test. Categorical variables are expressed as counts and percentages and were compared by the χ^2^ test. Kaplan–Meier analysis was used to assess the survival and incidence of clinical adverse events, and the log-rank test was used for initial comparisons. Prognostic value was tested using univariate and Cox proportional hazards analyses. In the subgroup of patients with Child–Pugh stage A, Kaplan–Meier analysis was performed to assess the survival and incidence of clinical events. The median values of the mean PAP, PVR, and cardiac index after medication were used as cutoff points for predicting adverse cardiac events. All statistical analyses were performed using SPSS version 29 software (IBM Corp., Armonk, NY, USA). Statistical significance was set at P < 0.05.

## 3 Results

### 3.1 Patient baseline characteristics

The baseline clinical characteristics of the study population are shown in Table 1. We enrolled 33 patients taking medications for PAH. In the total cohort, 10 patients (30 %) received upfront combination therapy and 23 (70 %) received initial monotherapy. Twenty-seven (82 %) patients were classified as Child–Pugh stage A, with a median Model for End-Stage Liver Disease (MELD) score of 10.0. We observed no significant differences in the baseline characteristics, hemodynamics, or exercise capacities between the monotherapy and upfront combination therapy groups. There was no liver transplantation candidate at baseline, and one liver transplantation candidate during follow-up.

**Table 1:** Baseline characteristics and hemodynamics.

Characteristic	All (n = 33)	Monotherapy (n = 23)	Upfront combination therapy (n = 10)	P value
Age, years	45 ± 16	46 ± 17	44 ± 13	0.653
Sex, female	23 (70 %)	16 (70 %)	7 (70 %)	0.980
Body mass index	24.3 ± 6.0	24.8 ± 6.3	22.9 ± 5.6	0.401
WHO functional class				0.235
Ⅱ	15 (45 %)	11 (48 %)	4 (40 %)	
Ⅲ	18 (55 %)	12 (52 %)	6 (60 %)	
Ⅳ	0	0	0	
Child–Pugh stage				0.817
A	27 (82 %)	19 (83 %)	8 (80 %)	
B	6 (18 %)	4 (17 %)	2 (20 %)	
C	0	0	0	
MELD score	10.0 (7, 12.5)	10.0 (7.0, 13.0)	9.5 (6.7, 11.2)	0.490
Brain natriuretic peptide, pg/mL	97 (27, 256)	95 (22, 300)	93 (35, 188)	0.985
6-min walking distance, m	378 (311, 430)	378 (305, 431)	379 (307, 446)	0.734
Etiology				
Liver cirrhosis	23 (70 %)	18 (78 %)	5 (50 %)	0.104
Hepatitis C	8 (24 %)	7 (30 %)	1 (10 %)	0.208
Alcoholic hepatitis	6 (18 %)	5 (22 %)	1 (10 %)	0.422
Autoimmune hepatitis	3 (9 %)	1 (4 %)	2 (20 %)	0.151
Primary biliary cirrhosis	2 (6 %)	2 (9 %)	0	0.336
Non-alcoholic steatohepatitis	1 (6 %)	1 (4 %)	0	0.503
Unknown	3 (9 %)	2 (9 %)	1 (10 %)	0.905
Congenital absence of the portal vein	4 (12 %)	3 (13 %)	1 (10 %)	0.806
Idiopathic portal hypertension	2 (6 %)	0	2 (20 %)	0.027
Congenital biliary atresia (post Kasai procedure)	3 (9 %)	2 (9 %)	1 (10 %)	0.905
Congenital portosystemic shunt	1 (3 %)	0	1 (10 %)	0.124
Hemodynamics				
Mean pulmonary artery pressure, mm Hg	43 (37, 52)	42 (36, 50)	47 (42, 54)	0.081
Pulmonary artery wedge pressure, mm Hg	5 (4, 8)	5 (4, 8)	6 (5, 7)	0.985
Mean right atrium pressure, mm Hg	5 (2, 7)	5 (1, 7)	4 (3, 7)	0.985
Cardiac index, L/min/m^2^	2.2 (1.9, 3.0)	2.2 (1.9, 2.7)	2.5 (2.1, 3.7)	0.123
Pulmonary vascular resistance, Wood units	10.4 (7.0, 14.1)	10.4 (6.8, 15.7)	9.9 (6.9, 13.1)	0.832
S_V_O_2_, %	68 (61, 73)	68 (59, 73)	68 (63, 74)	0.499
Medication				
*Monotherapy*				
Epoprostenol		4 (17 %)	–	
ERA		11 (48 %)	–	
PDE-5i		8 (35 %)	–	
*Upfront combination therapy*				
ERA + PDE-5i		–	9 (90 %)	
ERA + sGC stimulator		–	1 (10 %)	

Values are mean ± SD, median (interquartile range), or n (%). WHO, World Health Organization; MELD, Model for End-Stage Liver Disease; ERA, endothelin receptor antagonist; PDE-5i, phosphodiesterase type-5 inhibitor; sGC, soluble guanylate cyclase; S_V_O_2_, mixed venous oxygen saturations.

### 3.2 Change in hemodynamic parameters between monotherapy and upfront combination therapy

The average time at the first follow-up of RHC was 6.5 ± 1.4 months. The BNP, 6 MWD, mean PAP, cardiac index, and PVR significantly improved from baseline to follow-up in both the monotherapy and upfront combination therapy groups (Table 2). The change ratio of mean PAP (−16 % vs. −34 %, P < 0.001) and PVR (−32 % vs. −57 %, P = 0.006) was significantly better in patients who received upfront combination therapy than in those who received monotherapy. The WHO functional class significantly improved in the upfront combination group (P = 0.014) compared to the monotherapy group (P = 0.472). PAWP and right atrial pressure did not increase from baseline to follow-up in either group.

**Table 2:** Change in clinical and hemodynamic parameters from baseline to follow-up.

	Monotherapy (n = 20)			Upfront combination therapy (n = 10)			P value†
	Baseline	Follow-up	Change ratio (%)	Baseline	Follow-up	Change ratio (%)	
Brain natriuretic peptide, pg/mL	85 (23, 286)	35 (14, 69)∗	−27 (−82, −10)	93 (35, 188)	27 (22, 46)∗	−62 (−92, 13)	0.429
6-min walk distance, m	360 (290, 415)	420 (310, 460)∗	6 (1, 20)	379 (307, 446)	455 (380, 495)∗	23 (6, 29)	0.127
WHO-functional class (Ⅰ/Ⅱ/Ⅲ/Ⅳ)	0/8/12/0	1/12/7/0	–	0/4/6/0	3/7/0/0∗	–	–
Four-risk strata model (low/intermediate-low/intermediate-high/high)	2/6/9/2 (n = 19)	5/7/7/0∗(n = 19)	–	1/3/6/0	5/5/0/0∗	–	–
French invasive score (criteria 4/3/2/1/0)	1/3/4/10/1 (n = 19)	4/7/4/4/0 (n = 19)	–	1/2/3/3/1	7/2/1/0/0∗	–	–
Mean pulmonary artery pressure, mm Hg	40 (35, 51)	34 (30, 41)∗	−16 (−23, −5)	47 (42, 54)	33 (28, 37)∗	−34 (−47, −27)	<0.001
Pulmonary artery wedge pressure, mm Hg	6 (4, 8)	7 (5, 9)	23 (−15, 68)	6 (5, 7)	6 (5, 11)	20 (−4, 75)	0.846
Mean right atrium pressure, mm Hg	5 (1, 7)	3 (2, 6)	−36 (−53, 23)	4 (3, 7)	3 (2,5)	8 (−68, 37)	0.689
Cardiac index, L/min/m^2^	2.1 (1.9, 2.6)	2.6 (2.1, 3.1)∗	12 (−3, 55)	2.5 (2.1, 3.7)	3.7 (2.7, 4.1)∗	21 (7, 48)	0.373
Pulmonary vascular resistance, Wood units	10.2 (6.3, 15.4)	7.2 (4.0, 9.8)∗	−32 (−43, −25)	9.9 (6.9, 13.1)	4.6 (3.6, 6.5)∗	−57 (−67, −36)	0.006
S_V_O_2_, %	68 (57, 72)	67 (61, 72)	0 (−4, 11)	68 (63, 74)	75 (71, 78)∗	8 (1, 13)	0.164

Median (interquartile range).

∗ , P < 0.05, baseline vs. follow-up.

† , monotherapy vs. upfront combination therapy compared on change ratio; WHO, World Health Organization; S_V_O_2_, mixed venous oxygen saturations.

### 3.3 Changes in risk profiles after upfront combination therapy

**Fig. 1:**
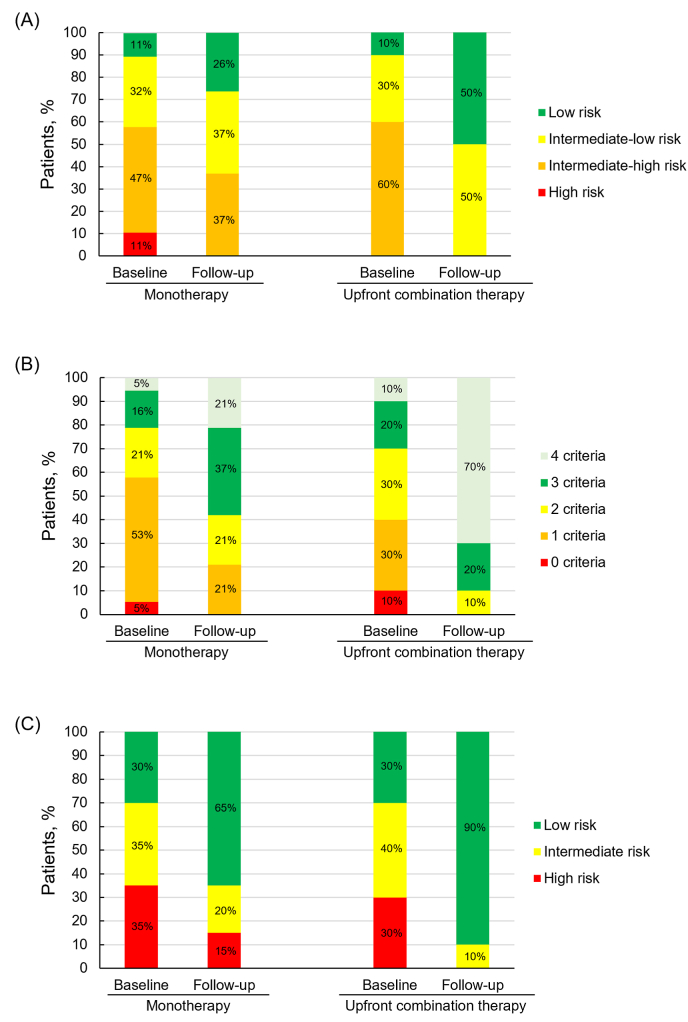
Evolution in risk profiles using the simplified 4-strata model (A),the French invasive score (B), and REVEAL Lite 2.0 score (C).

In the upfront combination therapy group, the proportion of patients achieving low or intermediate-low risk in the simplified four-strata risk assessment increased significantly from 40 % at baseline to 100 % at follow-up (P = 0.003) (Fig. 1A). This contrasted with the monotherapy group, in which the increase was from 33 % at baseline to 63 % at follow-up (P = 0.194). The proportion of patients who achieved a low-risk profile according to the invasive French method (three or four low-risk criteria) increased from 30 % at baseline to 90 % at follow-up in the upfront combination therapy group (P = 0.006), and from 21 % at baseline to 58 % at follow-up in the monotherapy group (P = 0.020) (Fig. 1B). REVEAL 2.0 Lite score demonstrated that low risk profiles increased from 30 % at baseline to 90 % at follow-up in the upfront combination therapy group (P = 0.020) and from 30 % at baseline to 65 % at follow-up in the monotherapy group (P = 0.140) (Fig. 1C).

### 3.4 Long-term effects of combined upfront combination therapy

**Fig. 2:**
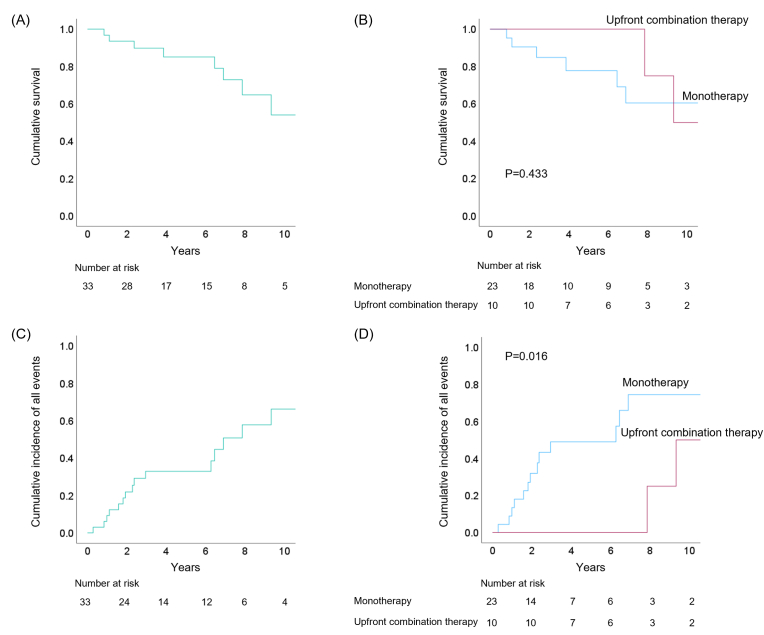
Kaplan–Meier analysis of survival and clinical event incidence. (A) Survival of the overall cohort of patients with portopulmonary hypertension (PoPH); (B) survival of patients with PoPH comparing monotherapy and upfront combination therapy; (C) cumulative incidence of clinical events in the overall cohort; (D) cumulative incidence of clinical events in patients with PoPH comparing monotherapy and upfront combination therapy.

Over a follow-up period of 5.6 ± 3.8 years, we observed 15 (46 %) adverse cardiac events. Of these, composite all-cause death occurred in seven patients, hospitalization for worsening PAH occurred in two patients, and six patients required additional PAH-specific medication due to worsening PH. Kaplan–Meier analysis was conducted to examine the overall survival and occurrence of clinical events in the entire group of patients with PoPH (Fig. 2A and C). The analysis revealed that the survival rates of patients with PoPH at 1, 3, 5, and 10 years were 94 %, 88 %, 83 %, and 56 %, respectively. Moreover, the analysis showed no significant difference in all-cause mortality between the patients who received monotherapy and those who received upfront combination therapy (log-rank = 0.433) (Fig. 2B). The incidence of clinical events was significantly lower in the patients who received upfront combination therapy (log-rank = 0.016) (Fig. 2D). In the univariate Cox proportional hazard analysis for clinical events in relation to pulmonary hypertension (Supplemental Table 1), upfront combination therapy was a factor for decreasing the composite of adverse outcomes (hazard ratio [HR] 0.190, 95 % confidence interval [CI] 0.042–0.854; P = 0.030). The median change ratio for PVR was −37 %, which was also a factor for predicting adverse cardiac events (HR 0.272, 95 % CI 0.084–0.878; P = 0.029). In the subgroup of patients with Child–Pugh stage A, the Kaplan–Meier analysis is demonstrated in Supplemental Fig. 2.

### 3.5 Safety of upfront combination therapy

The baseline levels of aspartate aminotransferase, alanine aminotransferase, and total bilirubin did not worsen following upfront combination therapy, as observed from the baseline to the two-year follow-up (Supplemental Fig. 3). Similarly, no significant changes in hemoglobin concentrations were observed. We observed no significant differences in adverse events between the monotherapy and upfront combination therapy groups (Supplemental Table 2). All patients continued PAH medication, and we observed no deaths related to PAH medication usage or gastrointestinal bleeding.

## 4 Discussion

In this study, upfront combination therapy for PoPH showed 1) better improvement of symptoms and hemodynamics compared to monotherapy; 2) significantly improved risk profiles and long-term outcomes in comparison with monotherapy; and 3) safety in terms of hepatic impairments and side effects. Upfront combination may be a treatment choice for patients with severe PoPH with mild hepatic impairments.

Upfront combination therapy showed long-term benefits in reduction of clinical worsening events compared to monotherapy and was a factor in decreasing the composite of clinical events. However, we observed no significant difference in long-term survival between the upfront combination therapy and monotherapy groups. Improvements in risk assessment scores have been associated with future clinical outcomes [16,17]. Upfront combination therapy also improved the risk profile, reaching approximately 90 % of the low-risk profile in our study. A better improvement of symptoms in upfront combination therapy than in monotherapy is important not only for clinical parameters but also for risk stratification [13,15]. These results also suggest benefits from upfront combination therapy in terms of long-term clinical outcomes. Although we observed no differences in the improvement of 6 MWD between upfront combination therapy and monotherapy, it is important to note that the improvement in exercise capacity is controversial in patients with PoPH taking PAH-specific drugs [3,5,6,12]. In addition, in patients presenting with mild liver disease, the main causes of death were PAH progression and malignancy, but in patients with advanced liver disease, complications of liver disease were the most common causes of death [13]. The small number of patients in our study may be a factor obscuring survival differences. We recommend upfront combination therapy to minimize clinical events related to pulmonary hypertension in patients with PoPH, particularly when liver impairment is mild.

Our study did not reveal any significant correlation between adverse events and MELD scores or Child–Pugh stage, despite previous research showing that MELD scores and Child–Pugh stage were independent risk factors for adverse events in patients with PoPH [3,20]. In our study, almost 80 % of the patients had Child–Pugh stage A, and the MELD score was low. It should be noted that the higher prevalence of mild hepatic impairment observed in our study may have contributed to these results. This contrasts with previous studies on PAH medication for patients with PoPH, which included only 40–50 % of patients at Child–Pugh A stage [3,5,6]. Importantly, the correlation is poor between the severity of PoPH and the degree of liver dysfunction and hepatic venous pressure gradient [21].

The upfront combination therapy was better than monotherapy in improving hemodynamics. A previous study demonstrated that PVR was an independent predictor of adverse events in patients with PoPH [22]. Early improvements in PVR were associated with future lung transplant-free survival in patients with PAH [23,24]. Furthermore, the importance of aggressive PVR reduction was recommended to improve right ventricular function and clinical outcomes [25]. The PORTICO study demonstrated the efficacy of macitentan versus placebo, with a 35 % reduction in PVR but limited improvement in exercise capacity. In contrast, in our study, upfront combination therapy achieved a 57 % reduction in PVR. Improvements in hemodynamic status are also a crucial goal in patients with PoPH for whom transplantation is being considered because severe pulmonary hypertension is a contraindication for liver transplantation [22,26]. Furthermore, the mean PAP decreased by 34 % in the upfront combination therapy group in our study, suggesting that this therapy could be a more potent treatment strategy for PoPH than monotherapy. Eight out of ten patients (80 %) achieved mean PAP <35 mm Hg or PVR <5 Wood units, which allows for potentially safer liver transplantation and satisfies the criteria to attain an increased MELD score for a higher liver transplant priority [27]. Upfront combination therapy thus represents a strategic option for aggressively improving hemodynamics.

Upfront combination therapy was safe in terms of hepatic function and side effects in the patients with mild hepatic impairment. The liver is the predominant metabolic site of PAH-specific medications, which therefore are expected to have a longer half-life and higher serum concentrations in patients with liver disease, leading to more frequent side effects. Liver dysfunction and fluid retention are the main side effects of vasodilator use in patients with PoPH [28]. Previous studies have also demonstrated the safety of monotherapy for PoPH [6,29,30]. Our findings, which show no significant hepatic dysfunction or serious adverse events with the use of upfront combination therapy, may partly be due to the relatively high proportion of patients with mild hepatic impairments in our sample. The differences in hepatic severity in patients with PoPH observed between countries may also be due to variability in screening systems and the prevalence of cirrhosis. Before determining treatment strategies, it is crucial to evaluate the liver condition and hemodynamic status of patients.

### 4.1 Limitations

The present study has several limitations. This was a single-center, retrospective study with a relatively small cohort of patients. The applicability of our findings across different populations and clinical settings is influenced by biases related to patient selection and data collection. Therefore, our study may have various confounding factors; for instance, the mean PAP appeared lower in the monotherapy group, which may have affected the conclusion of this study. Further prospective and randomized trials are needed to elucidate these biases and confounding factors and to clarify the influence of upfront combination therapy on clinical parameters and prognosis. Second, our study predominantly included young patients with Child–Pugh stage A disease and low MELD scores, making it difficult to generalize our findings to patients with severe or end-stage liver disease, particularly those in urgent need of liver transplantation. Larger populations are necessary to better understand the specific pathways and mechanisms involved.

### 4.2 Conclusion

Upfront combination therapy improved symptoms, hemodynamics, and clinical outcomes in patients with PoPH without serious adverse effects during the observation period. This study's findings suggest long-term benefits of upfront combination therapy in patients with PoPH presenting with mild hepatic impairment. Further large-scale study is warranted to confirm the effectiveness and safety of upfront combination therapy in patients with PoPH.

## CRediT authorship contribution statement

**Takatoyo Kiko:** Formal analysis, Visualization, Writing – original draft. **Ryotaro Asano:** Formal analysis, Writing – review & editing. **Hiroyuki Endo:** Data curation, Investigation. **Naruhiro Nishi:** Investigation. **Hiroya Hayashi:** Data curation. **Jin Ueda:** Investigation, Writing – review & editing. **Tatsuo Aoki:** Data curation, Investigation, Writing – review & editing. **Akihiro Tsuji:** Investigation, Writing – review & editing. **Takeshi Ogo:** Conceptualization, Formal analysis, Project administration, Supervision, Writing – review & editing.

## Declaration of competing interest

T. Ogo reports lecture fees from Nippon Shinyaku Co., Ltd.; Janssen Pharmaceutical K.K.; 10.13039/100015731Bayer Yakuhin, Ltd.; Nippon Shinyaku Co., Ltd.; GlaxoSmithKline K.K.; 10.13039/100010793Pfizer Japan Inc.; and Mochida Pharmaceutical Co., Ltd.; outside the submitted work.
