# Supplementary material for: Clinical outcomes of upfront combination therapy for portopulmonary hypertension
Source: Int J Cardiol Cardiovasc Risk Prev. 2024 May 31;22:200294. doi: 10.1016/j.ijcrp.2024.200294 (PMC11168483; doi:10.1016/j.ijcrp.2024.200294)
Supplement: Multimedia component 1 [file mmc1.docx]

**Supplemental figure 1.** Protocol of this study

**
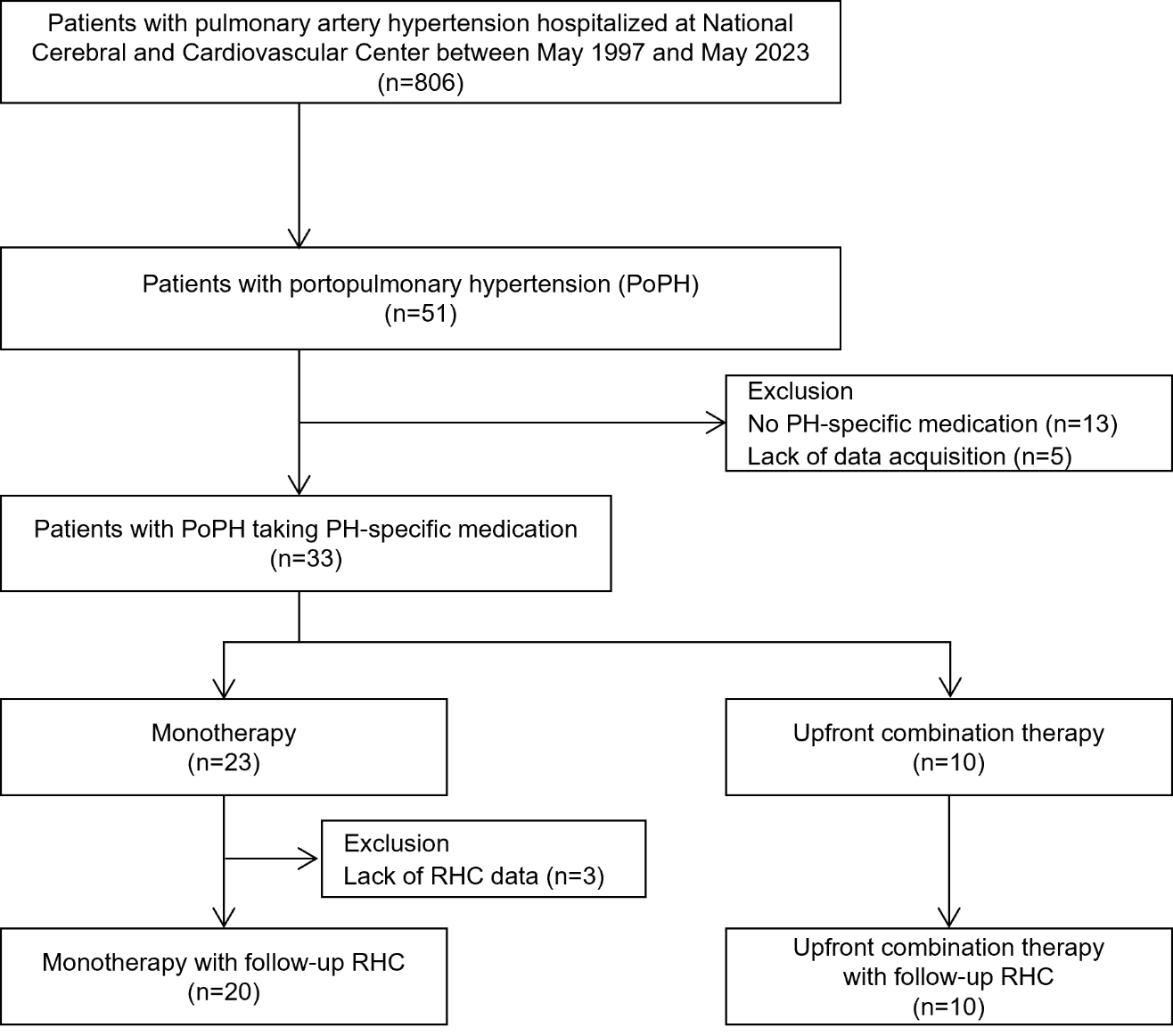
** PoPH, portopulmonary hypertension; PH, pulmonary hypertension; RHC, right heart catheterization**Supplemental figure 2.** Kaplan–Meier analysis of survival and clinical event incidence in the subgroup of patients with Child–Pugh A stage. (A) Survival of patients with PoPH comparing monotherapy and upfront combination therapy; (B) cumulative incidence of clinical events in patients with PoPH comparing monotherapy and upfront combination therapy.

PoPH, portopulmonary hypertension


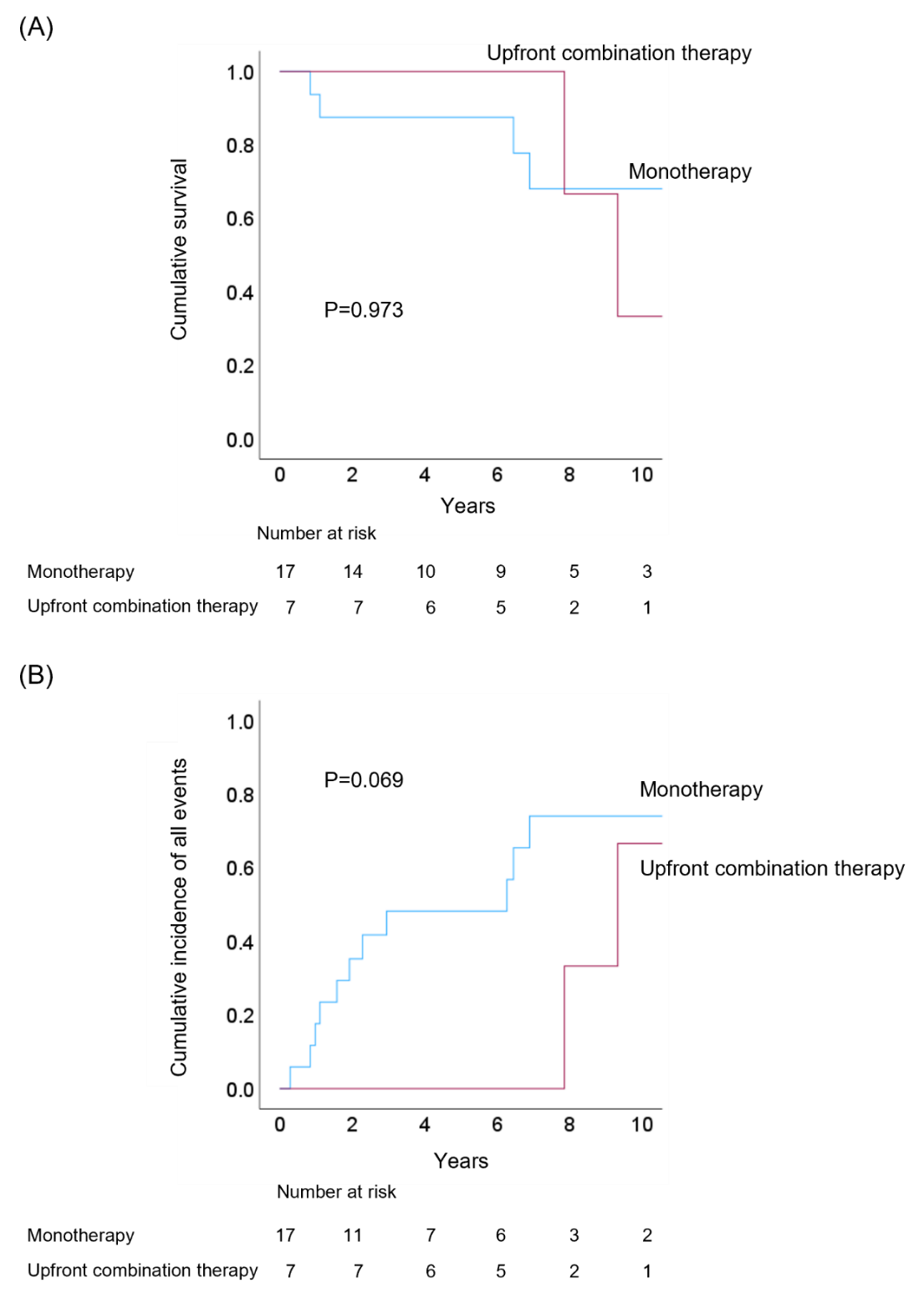


**Supplemental figure 3.** Safety of upfront combination therapy as assessed from liver function and hemoglobin concentration.


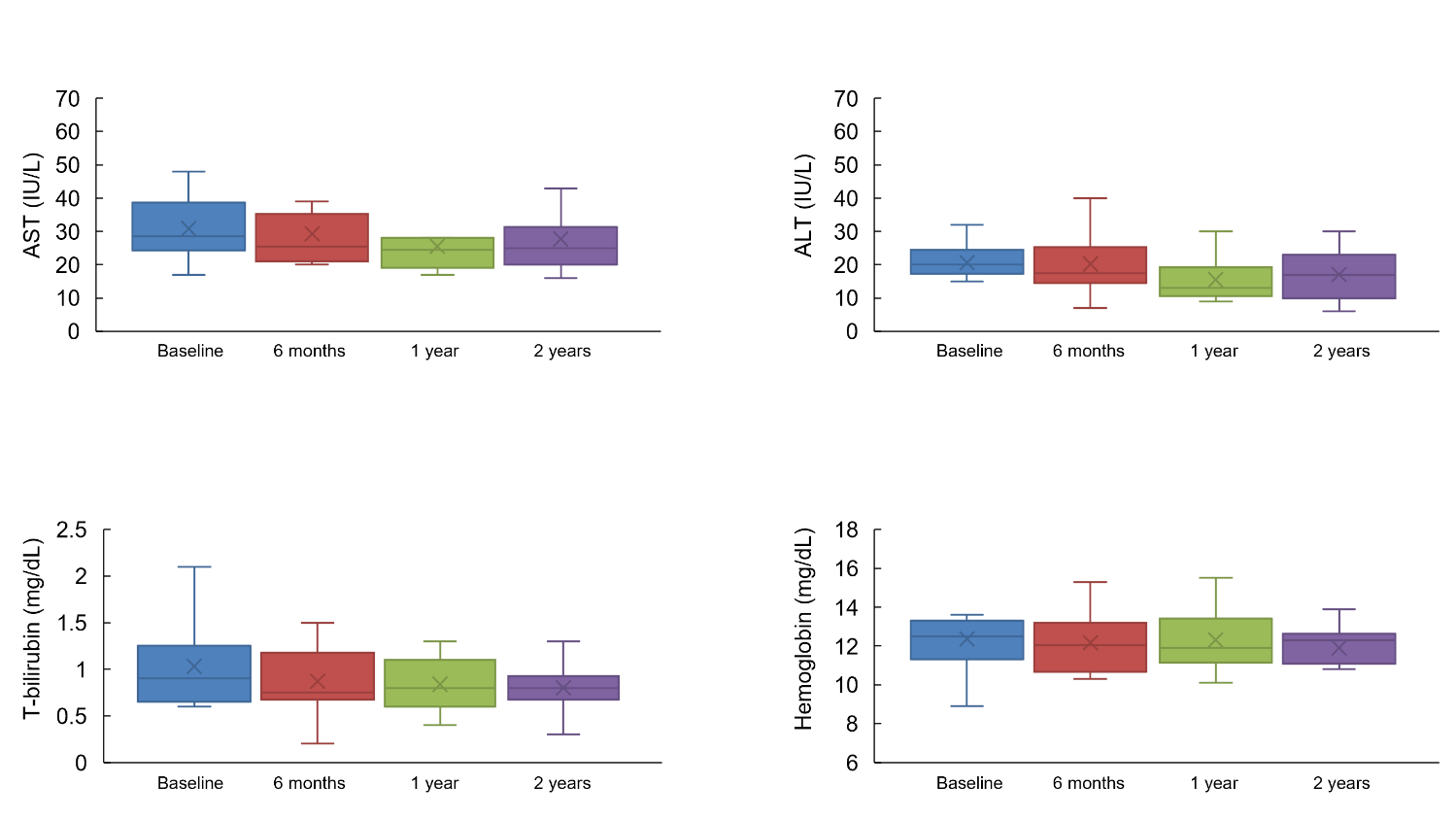
AST, aspartate aminotransferase; ALT, alanine aminotransferase
