# Supplementary material for: Clinical outcomes of upfront combination therapy for portopulmonary hypertension
Source: Int J Cardiol Cardiovasc Risk Prev. 2024 May 31;22:200294. doi: 10.1016/j.ijcrp.2024.200294 (PMC11168483; doi:10.1016/j.ijcrp.2024.200294)
Supplement: Multimedia component 2 [file mmc2.docx]

**Supplemental table 1.** Cox proportional hazard model for clinical events in relation to pulmonary hypertension

| Factor | Hazard ratio | 95% confidence interval | P value |
| --- | --- | --- | --- |
| Upfront combination therapy | 0.190 | 0.042–0.854 | 0.030 |
| Age | 0.997 | 0.967–1.028 | 0.835 |
| Sex, female | 2.617 | 0.586–11.685 | 0.208 |
| Child–Pugh stage | 0.555 | 0.124–2.494 | 0.443 |
| MELD score | 1.096 | 0.987–1.216 | 0.085 |
| Brain natriuretic peptide | 1.000 | 0.998–1.002 | 0.773 |
| 6-minute walking distance | 0.997 | 0.992–1.002 | 0.185 |
| *Etiology* |  |  |  |
| C hepatitis | 1.088 | 0.344–3.442 | 0.886 |
| Alcoholic hepatitis | 0.880 | 0.197–3.927 | 0.867 |
| Autoimmune hepatitis | 0.401 | 0.090–1.787 | 0.231 |
| Primary biliary cirrhosis | 5.701 | 0.286–5.760 | 0.031 |
| Non-alcoholic steatohepatitis | 30.984 | 1.937–495.534 | 0.015 |
| Congenital absence of the portal vein | 1.366 | 0.298–0.626 | 0.688 |
| Idiopathic portal hypertension | 0.042 | 0.001–86.327 | 0.415 |
| Congenital biliary atresia (post Kasai procedure) | 0.042 | 0.001–79.756 | 0.410 |
| Congenital portosystemic shunt | 0.969 | 0.123–7.650 | 0.976 |
| *Hemodynamics at baseline* |  |  |  |
| Mean pulmonary artery pressure | 1.004 | 0.968–1.041 | 0.839 |
| Pulmonary vascular resistance | 1.068 | 0.986–1.157 | 0.106 |
| Cardiac Index | 0.469 | 0.189–1.165 | 0.103 |
| *Change ratio of hemodynamics after medication* |  |  |  |
| change ratio of mean PAP <-21% (median) | 0.424 | 0.136–1.326 | 0.140 |
| change ratio of PVR <-37% (median) | 0.272 | 0.084–0.878 | 0.029 |
| change ratio of cardiac index >24% (median) | 1.063 | 0.354–3.193 | 0.913 |

PAP, pulmonary artery pressure; PVR, pulmonary vascular resistance

**Supplemental table 2.** Adverse events following medication in monotherapy and upfront combination therapy

| Adverse events | Monotherapy  (n = 23) | Upfront combination therapy  (n = 10) | P value |
| --- | --- | --- | --- |
| Serious adverse events | 0 | 0 | - |
| Non-serious adverse events |  |  |  |
| Headache | 8 (34.7) | 3 (30.0) | 0.789 |
| Peripheral edema | 3 (13.0) | 2 (20.0) | 0.609 |
| Flushing | 2 (8.6) | 1 (10.0) | 0.361 |
| Diarrhea | 2 (8.6) | 1 (10.0) | 0.361 |
| Lightheadedness | 3 (12.5) | 0 | 0.231 |
| Hypotension | 1 (4.3) | 1 (10.0) | 0.532 |
| Nausea | 1 (4.3) | 1 (10.0) | 0.532 |
| Nasal congestion | 2 (8.6) | 0 | 0.336 |
| Gastrointestinal bleeding | 0 | 0 | - |
